# Medical College and Hospital Notes

**Published:** 1896-05

**Authors:** 


### College and Hospital Notes.

It is stated in the newspaper press that land has been secured on Goodrich street for the erection of a new building, to be occupied as the College of Dentistry, which is the dental department of the University of Buffalo. The space the dental department now occupies in the old university building is badly needed by the medical department, which is also growing at a very rapid rate.

It is probable that ground will be broken almost immediately for the erection of the new building, which will be especially planned for the uses of a dental college of most advanced character. It will be able to accommodate a number of hundred students and will materially add to the educational advantages of Buffalo.

---

Dr. C. M. Daniels, chief surgeon of the Erie railway, announces the following plan for the care of the sick and disabled employees of that corporation : Hospitals will be established at convenient points along the entire system, including the N. Y., P. & O. and the Chicago & Atlantic. Wherever a hospital is already established it will be made a part of the new system. Membership in the organisation will be obligatory on all new employees, but with old ones it will not be compulsory, though it is believed they will generally avail themselves of its benefits.

The fund for the maintenance of the association will be derived from assessments. All officers, down to and including superintendents of divisions, will pay $1 a month ; employees receiving from $75 to $100 a month, 60 cents ; those receiving $50 a month, 50 cents ; those receiving less than $50, 25 cents a month ; while those whose monthly pay does not reach $25 will pay nothing. Membership in the association will confer upon the employees the right to go to the hospitals and remain as long as their illness continues, at the expense of the association.

It will give them the benefit of the best medical skill and hospital care and treatment free of cost to themselves. It is estimated that the annual revenues will be $105,000. The Erie company will contribute $10,000.

---

Dr. Woods Hutchinson, professor of anatomy in the State University of Iowa, has been chosen lecturer on Comparative Pathology and the Ancestry of Disease, in the Medical Department of the University of Buffalo.

